# Percieved Memory Decline and Advance Care Planning Engagement: Comparing LGBTQ+ and Non-LGBTQ+ Adults

**DOI:** 10.1093/geroni/igaf122.3993

**Published:** 2025-12-31

**Authors:** Olivia (YuHsuan) Wang, Mireille Jacobson

**Affiliations:** USC, Los Angeles, California, United States; University of Southern California, Los Angeles, California, United States

## Abstract

**Background:**

Advance care planning (ACP) allows individuals to discuss future medical decisions while cognitively capable. LGBTQ+ adults may benefit disproportionately, given weaker family support networks. They also report higher rates of perceived memory decline, but how cognitive concerns impact ACP and healthcare communication among LGBTQ+ adults remains understudied.

**Methods:**

We surveyed 2,398 adults (510 LGBTQ+) in the Understanding America Study, a national panel of U.S. adults aged 18+, to examine ACP preparedness and discussions in medical settings, and health care communication. Logistic regression models tested differences by LGBTQ+ status and interactions with perceived memory decline, controlling for demographics.

**Results:**

Compared to non-LGBTQ+ adults, LGBTQ+ adults were 3% more likely to have thought about ACP (p<.05) and 5% more likely to report ACP discussions in medical settings (p<.05). Among LGBTQ+ adults, perceived memory decline was associated with a higher likelihood of ACP discussions in medical settings compared with non-LGBTQ+ adults, and also with a greater likelihood of never having considered ACP. Perceived memory decline also reduced comfort with health care communication among LGBTQ+ adults.

**Conclusions:**

LGBTQ+ adults show greater ACP engagement overall, but perceived memory decline is linked to both higher likelihood of ACP discussions and higher likelihood of never having considered ACP. This suggests that ACP engagement among LGBTQ+ adults with perceived memory decline may be concentrated at different points along the planning spectrum. Providers should assess each patient’s starting point, validate perceived decline, and offer stage-appropriate interventions to foster communication and move patients toward timely ACP participation.

